# An assessment of agricultural pesticide use in Iran, 2012-2014

**DOI:** 10.1186/s40201-017-0272-4

**Published:** 2017-04-24

**Authors:** Zaim Morteza, Seyed Behzad Mousavi, Mohammad Ali Baghestani, Antero Aitio

**Affiliations:** 1 School of Public Health and Institute for Environmental Health Research, Teheran University of Medical Sciences, Tehran, Iran; 2 Plant Protection Organization, Islamic Republic of Iran, Tehran, Iran; 3 Finnish Institute of Occupational Health, Helsinki, Finland; 4 Department of Medical Entomology and Vector Control, School of Public Health, Tehran University of Medical Sciences, Pour Sina Street, PO Box 14155-6446, Tehran, Iran

**Keywords:** Pesticide management, Pesticide regulation, Pesticide use, Pesticide registration, Agriculture pesticides, Iran

## Abstract

**Background:**

This is the first published assessment on agricultural pesticide use in Iran with the aim to identify pesticide products with a potential of causing acute or chronic hazard to human health. It also establishes a baseline for future comparisons and for trend assessments.

**Methods:**

The amounts of imported technical materials for formulation by local manufacturers as well as imported final product formulations were extracted from the registration data of the Plant Protection Organization of Iran in 2012–2014. The hazard indicators used were acute oral toxicity and chronic toxicity. For the latter, carcinogenicity, mutagenicity, and adverse effects on reproduction or development (CMR toxicity), and low Acceptable Daily Intake (ADI) were used. The comparative potential of the active ingredients of concern in terms of chronic toxicity was assessed using the average annual volume of their estimated use divided by their respective ADI, called chronic hazard potential (CHP) in the present text. The contribution of individual pesticides in different use categories to the total CHP of the user category, was also calculated, using the average annual volume of the active ingredients of all pesticides used during the period 2012-2014.

**Results:**

On average about 14,000 tonnes of agriculture pesticides, expressed in active ingredients (AI), were annually used in Iran. Herbicides constituted the largest volume (43%), followed by insecticides and acaricides (37%) and fungicides (19%). 0.1% and 47% of the formulated products met the criteria of WHO Class Ib (highly hazardous) and Class II (moderately hazardous) products respectively. Aluminium phosphide and magnesium phosphide were identified as products of primary concern and chlorpyrifos, diazinon and paraquat as products of secondary concern, in terms of their acute human health hazard. No compound in carcinogenicity category 1A or 1B or germ cell mutagenicity/reproduction toxicity category 1A was identified. Six compounds (diazinon, chlorpyrifos, dichlorvos, metam sodium, paraquat and dimethoate) were identified as products with chronic hazard potential based on a low ADI.

**Conclusions:**

The assessment identified and prioritized agriculture pesticide used in Iran in terms of their acute and chronic hazard to human health for re-registration scheme recently established by PPO and for risk mitigation. It also set priority for research into development of alternative products and practices to minimize pesticide risks. Chronic hazard potential - amount of use adjusted with toxicity may serve as a useful point of reference for trend analysis also in the use of less hazardous agricultural pesticide products.

## Background

Iran (Islamic Republic of), with a total land area of 1,648,195 km^2^, lies between 25° 00’ and 39° 47’ N and 44° 02’ and 63° 20’ E. The country has on the northeast side, the desert and steppe of Turkmenistan and on the south and southwest side, the hot and arid Arabian Peninsula.

Of the total land area, some 90 million hectares (54.6%) are rangeland; 12.4 million hectares (7.5%) are forests; and 34 million hectares (20.6%) are deserts [1]. Cereals are grown on 70% of cultivated land (about 12 million hectares in 2014), with wheat - the country’s main staple - accounting for over half of total crop production [2]. Other important crops include potatoes, dates, figs, pistachios, walnuts, almonds, cotton, sugarcane, sugar beet, tea and tobacco. Fruit trees were cultivated on about 2.2 million hectares with about 17 million tonnes of yield in 2014 [3].

The Plant Protection Act, approved in 1966, covers the regulation of pesticide use in agriculture and the Plant Protection Organization (PPO) is the executive authority for the management of these chemicals in the country.

The objectives of this study were: (i) to review the pesticide use in Iran during period 2012 to 2014 and the potential human health hazards involved; (ii) to rank pesticides based on their human health hazards for prioritizing risk assessment and product review and re-registration; (iii) to establish a baseline for future comparisons and trend assessments; and (iv) to set priority for research to minimize pesticide exposure and risk. Use of pesticides for public health purposes (i.e. disease vector control pesticides, household pesticide products and professional pest control pesticides) is not considered. The regulation of these products is with Iran Food and Drug Administration and data on their importation and use is not available.

Hazard is the inherent property of a substance, such as a pesticide, to have a harmful effect on human health or environment, while risk is the probability of the occurrence and severity of such an adverse effect. Therefore, risk is a function of hazard times exposure. A hazard assessment is generally seen as a first step towards a full-fledged risk assessment. The evaluation of actual risks posed by pesticides would require location- and individual-specific exposure profiles that are beyond the scope of this assessment.

## Methods

The data on pesticide amounts used in this assessment are those registered by PPO and covered the years 2012 to 2014. Data on imported technical material (for formulation by local manufacturers in Iran) as well as imported product formulations were included. The registration of the data is compulsory and is required for an import permit by the PPO. It is assumed that the figures presented here are well representative of pesticide use in Iran.

The hazard indicators used were acute oral toxicity (i.e. the adverse effects occurring within a short time of administration of a single dose of a pesticide or immediately after short or continuous exposure of multiple doses over 24 h or less) and chronic toxicity including carcinogenicity, mutagenicity, adverse effects on reproduction or development (CMR toxicity) and marked long-term toxicity expressed as a low ADI.

Acute toxicity data were mainly gathered from the following sources: (i) The World Health Organization (WHO) recommended classification of pesticides by hazard - guidelines to classification 2009 [4] (ii) JMPR (Joint Meeting on Pesticide Residues) database [5] or (iii) EU Pesticides database [6].

The acute hazard classification of the pesticide formulations was determined based on the WHO recommended classification of pesticides by hazard [4]. Only acute oral toxicity was used as dermal toxicity data were available in the source for only few products. Furthermore, use of oral data in almost all cases results in a more strict hazard classification than dermal toxicity, and thus represents the worst case.

The criteria for acute hazard classification are shown in Table 1. Pesticide formulations containing a single active ingredient were classified based on the proportionate calculation of the acute oral LD_50_ of the active ingredient and the concentration of the active ingredient in the formulated product and the categories shown in Table 1. Toxicity data for mixture products were based on the acute oral toxicity of the pesticide formulation as registered by the PPO. The same assessment was conducted for products formulated in Iran (i.e. from the imported technical material), based on the type of formulation and concentration that was registered with PPO [4].

**Table 1:** WHO recommended classification of pesticides by hazard [4]

WHO Class		LD_50_ (mg/kg body weight)
		Oral
Ia	Extremely hazardous	<5
Ib	Highly hazardous	5-50
II	Moderately hazardous	50-2000
III	Slightly hazardous	2000-5000
U	Unlikely to present acute hazard	5000 or higher

Formulated products meeting the criteria of WHO hazard class Ia or Ib were considered of primary concern; products with toxicity in the class II, and with at least 5% of total pesticide use, were considered as secondary concern.

For chronic toxicity, products that were classified by European Food Safety Authority (EFSA) for carcinogenicity (1 or 2), mutagenicity (1A, 1B or 2), or toxic to reproduction (1A, 1B or 2) as well products with an ADI of lower than 0.005 were studied [6]. The limit, 0.005 mg/kg body weight is arbitrary but represents a limit below which only about 10% of all pesticides approved in the EU fall. The EU classification and EFSA ADI were selected, as the information was available for most pesticides used in Iran and thus allowing use of constant criteria for all pesticides – in addition to being representative of classifications and assessments based on transparent and well-defined procedure and utilization of all data (published and non-published).

To compare chronic hazards involved with the use of individual pesticides, a parameter “chronic hazard potential” (CHP) was developed: average annual use divided by the ADI. For each pesticide, its contribution to the total pesticide chronic hazard potential was also calculated.

## Results

On average, close to 11,000 tonnes of active ingredient of pesticides in the form of technical material was annually imported to Iran for formulation by local pesticide manufacturers during 2012-2014 (Table 2). Technical material of herbicides constituted the largest number and volume of import (42 active ingredients and 4,918 tonnes, i.e. 45%), followed by that of insecticides and acaricides (35 active ingredients and 4,370 tonnes, i.e. 41%). The herbicides paraquat, glyphosate and butachlor, and the insecticides, diazinon and chlorpyrifos constituted about half of the total import of technical materials, respectively.

**Table 2:** Number and average annual volume of technical material (AI) of pesticides imported to Iran during 2012-2014

Use category	No. of technical material (AI)	Volume (tonnes)
Herbicide	42	4918
Fungicide	22	1419
Insecticide & acaricide	35	4470
Rodenticide	3	0.007
Nematicide	1	325
Molluscicide	1	24
Total	104	10831

On average, about 3,000 tonnes of active ingredients of pesticides in the form of formulated products (either as single active ingredient (AI) or as a mixture of active ingredients) were annually imported into Iran during 2012-2014 (Table 3). Fungicides constituted the largest volume of total import (1199 tonnes AI, i.e. 38%), followed by herbicides (1097 tonnes AI, i.e. 35%) and insecticides & acaricides (788 tonnes AI, i.e. 25%). Mancozeb (fungicide) and glyphosate (herbicide) constituted about one-third of the average annual import of single active ingredient formulated products.

**Table 3:** Number and volume of agriculture pesticide formulated products imported to Iran during 2012-2014

Use category	Product	No. of products	Average annual volume (tonnes of AI^a^)
Herbicide	Single AI	36	916
	Mixture AI	14	181
Fungicide	Single AI	25	1139
	Mixture AI	13	60
Insecticide & acaricide	Single AI	45	785
	Mixture AI	3	2,8
Rodenticide	Single AI	5	16
Other^b^	Single AI	6	23
Sub-total	Single AI	116	2889
	Mixture AI	30	244
Grand total			3133

^a^ *AI* active ingredient

^b^Includes 2014 importation of 30.4 tonnes (annual average 10.1 tonnes) methyl bromide for use in quarantine of imported plant products into Iran

**Fig. 1:**
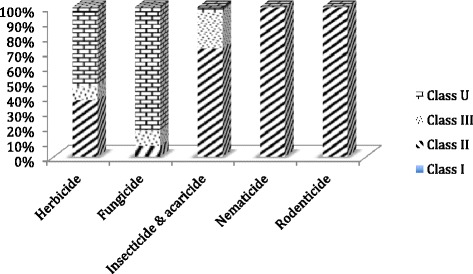
WHO hazard classification of imported and manufactured agriculture pesticides in Iran during 2012-2014 based on main use categories. Class I (Ia, extremely hazardous; Ib, highly hazardous); class II (moderately hazardous); class III (slightly hazardous); and class U (unlikely to present acute hazard)

Of the total average annual volume of imported pesticides into Iran during 2012-2014, either as technical material for formulation in the country, or as ready to use products, only 0.1% of the formulated products met the criteria of WHO Class Ib highly hazardous products (Table 4). About 47% of the products fell under WHO Class II (moderately hazardous), while remaining met the criteria of WHO Class III (slightly hazardous) or U (unlikely to present acute hazard in normal use). Figure 1 presents the WHO hazard classification of the main pesticide use categories in Iran during 2012-2014. WHO class II formulated pesticide products included importation of 30,380 kg of methyl bromide gas in 2014 for use in quarantine of imported plant products into Iran or of those to be exported. Under Montreal Protocol on Substances that Deplete Ozone Layer [7] the use of this product has significantly been reduced in Iran in recent years. Noting the physical nature and restricted use of this compound, it has not been included in chronic hazard assessment presented below.

**Table 4:** Amount of pesticide products imported to or manufactured in Iran, per WHO class^a^ of acute toxicity

Use category	Tonnes of active ingredient per year			
	Class I	Class II	Class III	Class U
Herbicide	0	2271	559	3184
Fungicide	0	165	245	1884
Insecticide & acaricide	10	3789	1288	171
Rodenticide	0	16	0	0.008
Nematicide	0	341	0	0
Other	0	10	31	0.2
Total	10	6593	2122	5239

^a^Class I (Ia, extremely hazardous; Ib, highly hazardous); class II (moderately hazardous); class III (slightly hazardous); and class U (unlikely to present acute hazard)

The only products in WHO group I were aluminium phosphide and magnesium phosphide, used mainly as insecticides for the control of grain store pests, but also occasionally as a rodenticides and thus considered of primary concern in terms of acute human health hazard (see Table 5). Forty-two products met the WHO criteria of Class II, namely the herbicides 4, the fungicides 10, the insecticides & acaricides 26, the rodenticides 1 and the nematicides 1. Of these, only the insecticide products chlorpyrifos and diazinon, and the herbicide product paraquat, met our criteria of secondary concern (5% of total pesticide use). They constituted 8, 11 and 9% of the total volume of pesticide formulations imported/manufactured in Iran during the 3 year period, respectively.

**Table 5:** Agriculture pesticide products of primary and secondary concern in terms of acute human health hazard, Iran 2012-2014

	Product	Use category	Percent of total average annual import of pesticides (in AI^a^)
Primary concern	Aluminium phosphide	Insecticide^b^	0.1
	Magnesium phosphide	Insecticide^b^	<0.1
Secondary concern	Chlorpyrifos	Insecticide	8
	Diazinon	Insecticide	6
	Paraquat	Herbicide	9

^a^ *AI* active ingredient

^b^Fumigant, also used occasionally as rodenticide

### Chronic hazard potential

Ten active ingredients sold in Iran have been classified by EFSA as potential carcinogens (category Carc 2). Two active ingredients have been classified in category 1B and one in category 2 for mutagenicity (M1B and M2). Three active ingredients have been classified in category 1B and 11 in category 2 for reproductive toxicity (R1B and R2) [6, 8]. Seventeen active ingredients had an ADI lower than 0.005 mg/kg.

Of these pesticides, which are candidates for products of concern, captan (C2), penconazole (R2) ioxynil (R2), molinate (R2,C2), spirotetramat (R2), bromoxynil (R2), epoxiconazole (C2), kresoxim methyl (C2), cymoxanil (R2), cycloxydim (R2), metazachlor (C2), terbutylazine (low ADI), and fenoxycarb (C2), contributed less than 0.01% each to the total pesticide-related chronic hazard potential (based on ADI and volume of use, see methods), and are excluded from the analysis of chronic hazard potential. Details on the 28 pesticides considered to present chronic hazard potential, are presented in Table 6.

**Table 6:** Active ingredients of chronic hazard potential

Active ingredient^a^	Toxic hazard^b^	ADI^c^	Annual use (tonnes)	Proportional use^d^	Chronic hazard potential^e^	Contribution to total CHP %^f^
Fungicides						
Benomyl	M1B, R1B	0.1^j^	155.3	2.2	1.6	0.015
Carbendazim	M1B, R1B	0.02	170.4	2.4	8.5	0.080
Chlorothalonil	C2	0.015	165.5	2.3	11.0	0.10
Iprodione	C2	0.06	110.6	1.6	1.8	0.017
Mancozeb	R2	0.05	790.0	11.2	15.8	0.15
Tebuconazole	R2	0.03	43.3	0.6	1.4	0.014
Thiophanate-methyl	M2	0.08	112.3	1.6	1.4	0.013
Tridemorph	R1B	0.016^j^	32.0	0.5	2.0	0.019
Insecticides/acaricides						
Abamectin	R2	0.0025	178.4	0.3	7.1	0.067
Azocyclotin	ADI	0.003	10.9	0.2	3.6	0.034
Chlorpyrifos	ADI	0.001	1134.3	16.0	1130	10.7
Diazinon	ADI	0.0002	1507.6	21.2	7540	70.7
Dichlorvos	ADI	0.00008	62.9	0.9	786	7.4
Dimethoate	ADI	0.001	166.2	2.4	166	1.6
Ethion	ADI	0.002	204.9	2.9	102	1.0
Fipronil	ADI	0.0002	0.8	0.01	4.2	0.039
Oxydemeton-methyl	ADI	0.0003	220.8	0.3	73.6	0.7
Propargite	C2	0.01^h^	183.5	2.6	18.4	0.17
Herbicides						
Acetochlor	ADI	0.0036	568.5	0.8	15.8	0.15
Clodinafob propargil	ADI	0.003	66.4	0.9	22.1	0.21
Diclofop-methyl	ADI	0.001	4.2	0.1	4.2	0.039
Haloxyfop r methyl	ADI	0.00065	439.0	0.6	67.5	0.6
Linuron	R1B, C2	0.003	13.7	0.2	4.6	0.043
Oxadiazon	ADI	0.0036	13.0	0.2	3.6	0.034
Oxyfluorfen	ADI	0.003	196.5	0.3	6.6	0.061
Paraquat	ADI	0.004	1179.6	16.7	295	2.8
Trifluralin	C2	0.015	314.7	4.4	21.0	0.20
Nematicide						
Metam sodium	ADI	0.001	341.6	4.8	342	3.2

^a^ *AI* insecticide and or acaricide, *N* nematicide, *H* herbicide, *F* fungicide

^b^Toxic Hazard: ADI = ADI < 0.005 mg/kg body weight; C2 = Carcinogenicity class 2 (EU); M1B = Mutagenicity class 1B (EU); R1B = Reproduction toxicity class 1B (EU); R2 = Reproduction toxicity class 2 (EU), M2 = Mutagenicity class 2 (EU)

^c^Acceptable daily intake from EFSA [6], if not otherwise stated

^d^Proportion of total use of potentially hazardous (long-term) products (CMR products, products with an ADI < 0.005)

^e^Average (annual use/ADI) ×10^-12^

^f^Proportion of total CHP due to potentially hazardous (long-term) products (CMR products, products with an ADI < 0.005) in per cent

^j^ ADI from Pesticide Safety Directorate, UK [16]

^h^ADI from JMPR [17]

Included are pesticides used in Iran, which the EFSA has classified as CMR hazards or for which the ADI has been set at ≤ 0.005 mg/kg body weight and which contribute more than 0.01% to the total chronic hazard potential of pesticides used in Iran

The chronic hazard potential (CHP) is dominated by the insecticides/acaricides diazinon, chlorpyrifos, dichlorvos, and the nematicide metam sodium, which contribute more than 90% to the total CHP; diazinon alone presents 71% of the total CHP. In addition, some diazinon products contain several very toxic impurities, some of which may also be generated upon storage. The impurities may considerably increase the toxicity of diazinon [9]. It is therefore of utmost importance to control the concentrations of the impurities in saleable diazinon. The contribution of chlorpyrifos to the total CHP is 10%. For chlorpyrifos is also important to control the concentration of the toxic impurity sulfotep [10]. Diazinon and chlorpyrifos were also identified as items of secondary concern for acute toxicity (See above). Dichlorvos was not approved to the Annex I of the European Union because of uncertainties on the carcinogenicity and mutagenicity [6]. Of the insecticides/acaricides only fenoxycarb, spirotetramat, propargite and abamectin were CMR classified.

Metam sodium, paraquat, dimethoate and ethion contributed 3.2, 2.8, 1.6, and 1.0% each to the total CHP, while the contribution of the other products was clearly below 1%. One should also note that the highest-ranking CMR product, trifluralin (C2), was not among the top ten, and among the CMR products, only trifluralin, propargite, mancozeb, and chlorothalonil had a contribution to the total CHP of more than 0.1%.

The fungicides all had a CMR classification, but only contributed 0.4% to the total CHP of the pesticides. Of the individual fungicides, the major contributors to the CHP were mancozeb (35% of the CHP of fungicides), chlorothalonil (24%), and carbendazim (19%).

Of the herbicides, bromoxynil, cycloxydim, ioxynil, linuron, metazachlor, molinate and trifluralin had a CMR classification; the herbicides contributed 4% to the total pesticide CHP. This was mainly due to paraquat (67%), haloxyfop r-methyl (15%), trifluralin (5%), and acetochlor (4%).

## Discussion

This is the first published report on agriculture pesticide use in Iran with the aim to identify and prioritize pesticide products of acute and chronic health hazard to human health for risk mitigation. It also establishes a baseline for future comparisons and for trend assessments.

About 14,000 tonnes of agricultural pesticides, expressed in active ingredient, was on average imported to, or manufactured in Iran annually during 2012-2014. Only two, 0.1% of their formulations belonged to WHO Class Ib highly hazardous pesticide products. These were aluminium phosphide and magnesium phosphide, highly toxic compounds, used for fumigation against stored product insects and occasionally rodents.

The International Code of Conduct on Pesticide Management urges governments “prohibition of the importation, distribution, sale and purchase of highly hazardous pesticides may be considered if, based on risk assessment, risk mitigation measures or good marketing practices are insufficient to ensure that the product can be handled without unacceptable risk to humans and the environment” [11]. Acute poisoning due to aluminium phosphide has been rather common in Iran. More than 950 cases of acute poisoning with this chemical were admitted during 2007–2010 to Loghman Hakim Hospital Poison Centre in Tehran - a referral center for poisoning for a population of about 14 millions - with a 24% mortality rate [12]. To address this issue, PPO in 2010 restricted the use of aluminium phosphide to the control of the quarantine pest, red palm weevil (*Rhynchophorus ferrugineus*) and the control of the rodent, *Spermophilus fulvus.* The new regulation however has not resulted in the decrease of the incidence of acute poisoning cases [13]. The incidence of fatal aluminium phosphide poisoning cases referred to the Legal Medicine Organization of Iran was 5.22 and 37.02 per million of population of Tehran in 2006 and 2013, respectively. Research into alternative approaches/products for the control of the above-mentioned agriculture pests, improved regulation especially in control of illegal import of aluminium phosphide to Iran, and public education on the toxicity of this compound are of high priority.

This study also identified the insecticides, chlorpyrifos and diazinon, and the herbicide paraquat, of special concern in terms of their acute human health hazards. The formulated products of the named compounds belonged to the WHO Class II and constituted in total about 28% of the average annual import of pesticides into Iran (in terms of active ingredient).

Acute toxicity occurs after short-term exposure to a pesticide and is especially relevant for pesticide applicators, pesticide loaders and workers who are cleaning equipment or storage sites. Proper risk mitigation measures and good marketing practices are necessary to minimize exposure to and acute risk associated with handling and use of pesticides. This, among other things, requires ensuring that persons involved in the sale of pesticides are adequately trained, holding appropriate license and having access to sufficient information so that they are capable of providing farmers with advice on proper handling and use of these chemicals. It is noteworthy that in 2015 about one-third of the 6532 pesticide stores in Iran lacked the appropriate license from the national regulatory authority (PPO, personal communication).

Human and environmental health impact of pesticide use in Iran has not been properly investigated. In a recent review of 57 published environmental and population studies concerning exposure to pesticides in Iran during the period 1960–2012 however has revealed alarmingly high levels of pesticide residues in the environment and of chronic and acute pesticide poisonings [14].

The present study collated data on the CMR toxicity, and on marked overall long-term toxicity of the pesticides used in Iran. No products classified as carcinogen category 1 (known or presumed to be carcinogenic to humans), reproductive toxicant category 1A (known human reproductive toxicant), or germ cell mutagens category 1A (known to induce heritable mutations in germ cells of humans) [8] were identified. Two pesticides used, carbendazim and benomyl, have classified in germ cell mutagenicity category 1B (substances which should be regarded as if they induce heritable mutations in the germ cells of humans), three (carbendazim, benomyl, linuron) in reproductive toxicant category 1B (presumed human reproductive toxicant). Eleven pesticides were classified in the carcinogenicity category 2 (suspected human carcinogens), 1 (thiophanate-methyl) in the germ cell mutagenicity category 2 (substances which cause concern for humans owing to the possibility that they may induce heritable mutations in the germ cells of humans), and 11 products in the reproductive toxicity category 2 (suspected human reproductive toxicant).

The CMR classification of a product was loosely related to the ADI determined by the same organization, EFSA: for all pesticides for which an EFSA ADI was available, the average ADI was 0.116 mg/kg body weight, that for pesticides classified based on carcinogenicity, mutagenicity, and reprotoxicity, 0.068, 0.067, and 0.029 mg/kg. On the other hand, for the pesticides selected in the present study for their chronic hazard potential, the average ADI was 0.002. The relatively small difference in the ADI of CMR and non-CMR pesticides probably reflects the fact that for most suspected CMR chemicals, CMR effects are observed at relatively high dose levels and other toxicity determines the ADI.

The present assessment identified 6 pesticide active ingredients that contribute >0.5% of the total chronic hazard potential (diazinon,chlorpyrifos, dichlorvos, metam sodium, paraquat, dimethoate), of which diazinon, chlorpyrifos and dichlorvos contributed, almost 90%. Promoting use of less hazardous alternative pesticides, application of best agricultural and pesticide application practices and effective regulation of pesticide residues in or on food are all important factors contributing to the reduction of risk associated with the use of these pesticides.

The International Code of Conduct on Pesticide Management urges governments to establish a re-registration procedure to ensure the regular review of pesticides, thus ensuring that prompt and effective measures can be taken if new information or data on the performance or risks indicate that regulatory action is needed [11]. Compounds of concern identified in this assessment should receive priority for such a review in Iran. A plan for review and re-registration of pesticides is underway by PPO.

Pesticide registration in Iran has been based on the assessment of the formulated products, while it is critical to link the hazard of the product to the technical material used in its formulation. Different manufacturing processes that are used by different suppliers may affect the purity/impurity profile of a technical material and therefore its hazard. PPO uses Food and Agriculture Organization of the United Nations (FAO) specifications, where available, for quality control of pesticides in Iran. It should be noted however that FAO specifications for formulations developed under the new procedure, unless otherwise stated, encompass the products of only those formulators that their products contain only active ingredient sourced from a manufacturer to whom the FAO specification for technical material applies [15]. It is also noteworthy that only 3 of the 6 pesticide compounds identified as of concern in terms of chronic toxicity in Iran (chlorpyrifos, dimethoate, paraquat) have FAO specifications under the new procedure [10]. PPO should follow the recommendations of the International Code of Conduct on Pesticide Management in implementing the principles described in the Manual on development and use of FAO and WHO specifications for pesticides to assess hazard and quality of pesticides in Iran [15]. Clearly, PPO should also keep abreast of the developments in international and other national organizations: for example, the EU has not approved the use of diazinon or dichlorvos, and has specifically limited the concentration of the major impurity, sulfotep in chlorpyrifos [6]. Paraquat, ethion and oxydemethon-methyl are not approved in the European Union [6]. This would require further strengthening of PPO, especially in the areas of toxicology and risk assessment of pesticides.

## Conclusions

The assessment identified agriculture pesticide use in Iran in terms of their acute and chronic hazard to human health. The information is important for prioritizing products for re-registration (a scheme has recently been established by PPO) and for risk mitigation. The assessment also sets priority for research into development of alternative products and practices, with the aim to minimize adverse effects on human health and the environment. Chronic hazard potential – a toxicity-adjusted use estimate, serves as a good point of reference for trend analysis in use of less hazardous agricultural pesticide products.

## Abbreviations

ADI: Acceptable daily intake
AI: Active ingredient
CHP: Chronic hazard potential
CMR: Carcinogenicity, mutagenicity, and adverse effects on reproduction or development
EFSA: European food safety authority
EU: European commission
FAO: Food and agriculture organization of the united nations
JMPR: Joint FAO/WHO meeting on pesticide residues
PPO: Plant protection organization (Iran)
WHO: World Health Organization
